# Neurally adjusted ventilatory assist vs. pressure support to deliver protective mechanical ventilation in patients with acute respiratory distress syndrome: a randomized crossover trial

**DOI:** 10.1186/s13613-020-0638-0

**Published:** 2020-02-10

**Authors:** Fabia Diniz-Silva, Henrique T. Moriya, Adriano M. Alencar, Marcelo B. P. Amato, Carlos R. R. Carvalho, Juliana C. Ferreira

**Affiliations:** 1 Divisao de Pneumologia, Instituto do Coracao, Hospital das Clinicas HCFMUSP, Faculdade de Medicina, Universidade de Sao Paulo, SP, BR, Av. Dr. Enéas de Carvalho Aguiar, 44, 5 andar, bloco 2, sala 1, São Paulo, SP CEP 05403900 Brazil; 2 Biomedical Engineering Laboratory, Escola Politécnica da USP, Av. Prof. Luciano Gualberto, trav. 3, 158, Cidade Universitária, São Paulo, SP CEP 05586-0600 Brazil; 3 Instituto de Física, Universidade de São Paulo, Caixa Postal 66318, São Paulo, SP CEP 05314-970 Brazil

**Keywords:** Respiration, artificial, Respiratory distress syndrome, adult, Interactive ventilatory support, Positive-pressure respiration, Neurally adjusted ventilatory assist

## Abstract

**Background:**

Protective mechanical ventilation is recommended for patients with acute respiratory distress syndrome (ARDS), but it usually requires controlled ventilation and sedation. Using neurally adjusted ventilatory assist (NAVA) or pressure support ventilation (PSV) could have additional benefits, including the use of lower sedative doses, improved patient–ventilator interaction and shortened duration of mechanical ventilation. We designed a pilot study to assess the feasibility of keeping tidal volume (*V*_T_) at protective levels with NAVA and PSV in patients with ARDS.

**Methods:**

We conducted a prospective randomized crossover trial in five ICUs from a university hospital in Brazil and included patients with ARDS transitioning from controlled ventilation to partial ventilatory support. NAVA and PSV were applied in random order, for 15 min each, followed by 3 h in NAVA. Flow, peak airway pressure (Paw) and electrical activity of the diaphragm (EAdi) were captured from the ventilator, and a software (Matlab, Mathworks, USA), automatically detected inspiratory efforts and calculated respiratory rate (RR) and *V*_T_. Asynchrony events detection was based on waveform analysis.

**Results:**

We randomized 20 patients, but the protocol was interrupted for five (25%) patients for whom we were unable to maintain *V*_T_ below 6.5 mL/kg in PSV due to strong inspiratory efforts and for one patient for whom we could not detect EAdi signal. For the 14 patients who completed the protocol, *V*_T_ was 5.8 ± 1.1 mL/kg for NAVA and 5.6 ± 1.0 mL/kg for PSV (*p* = 0.455) and there were no differences in RR (24 ± 7 for NAVA and 23 ± 7 for PSV, *p* = 0.661). Paw was greater in NAVA (21 ± 3 cmH_2_O) than in PSV (19 ± 3 cmH_2_O, *p* = 0.001). Most patients were under continuous sedation during the study. NAVA reduced triggering delay compared to PSV (*p* = 0.020) and the median asynchrony Index was 0.7% (0–2.7) in PSV and 0% (0–2.2) in NAVA (*p* = 0.6835).

**Conclusions:**

It was feasible to keep *V*_T_ in protective levels with NAVA and PSV for 75% of the patients. NAVA resulted in similar *V*_T_, RR and Paw compared to PSV. Our findings suggest that partial ventilatory assistance with NAVA and PSV is feasible as a protective ventilation strategy in selected ARDS patients under continuous sedation.

*Trial registration* ClinicalTrials.gov (NCT01519258). Registered 26 January 2012, https://clinicaltrials.gov/ct2/show/NCT01519258

## Background

Acute respiratory distress syndrome (ARDS) has a high mortality burden [1], especially in low and middle-income countries [2, 3]. Protective mechanical ventilation (MV)—consisting of the use of tidal volume (*V*_T_) equal or less than 6 mL/kg of predicted body weight (PBW) and plateau pressure (Pplat) limited to 30 cmH_2_O—reduces mortality and is recommended for ARDS [4–8]. In the initial phase of ARDS, patients are often ventilated with controlled modes for rigorous control of *V*_T_ and Pplat, requiring sedation and sometimes, neuromuscular blockade [6, 9–12], which are associated with diaphragmatic weakness [13–15]. On the other hand, overload of the respiratory muscles during acute respiratory failure causes muscle fatigue and is also associated with adverse events [16].

Using partial ventilatory support could be one alternative to prevent respiratory muscles weakness and complications associated with controlled mechanical ventilation [13, 14] while also preventing muscle fatigue [16]. Partial ventilatory support in ARDS could have additional benefits, including the use of lower sedative doses, improved patient–ventilator interaction and shortened duration of MV [12, 17, 18].

Neurally adjusted ventilatory assist (NAVA) is a proportional ventilatory mode that uses the electrical activity of the diaphragm (EAdi) to trigger, cycle and provide inspiratory assistance in proportion to patient’s effort [19–21]. Studies have shown that NAVA prevents excessive lung distension, reduces the work of breathing and improves patient–ventilator synchrony when compared with pressure support ventilation (PSV) [22–28].

We designed a pilot study to assess the feasibility of using NAVA and PSV for ARDS patients transitioning from controlled ventilation to partial ventilatory support. We hypothesized that it would be feasible to keep *V*_T_ at protective levels with NAVA and PSV.

## Methods

We conducted a crossover study in five intensive care units of a university hospital in São Paulo, Brazil, from November 2012 to November 2015. The Institution’s Ethics committee (CaPPesq 02874612.6.0000.0068) approved the study and informed consent was obtained from the families of patients and attending physicians. The study was registered on ClinicalTrials.gov (NCT01519258).

We screened intubated and mechanically ventilated patients with ARDS according to the Berlin definition [29] and included a pilot sample of 20 patients. We could not calculate a sample size since data on the performance of NAVA during protective ventilation for ARDS was not available in the literature. Inclusion criteria were MV for more than 24 h; diagnosis of ARDS; indication of protective MV, by the ICU team; presence of inspiratory efforts triggering the ventilator for more than 6 h. Exclusion criteria were age < 18 years, pregnancy, severe hemodynamic instability, contraindications to the placement of the esophageal catheter and participation in other clinical trials.

We randomized patients who fulfilled all inclusion criteria and no exclusion criteria to the order of ventilation in NAVA and PSV using a computer generated randomization list (http://www.R-project.org/, Vienna, Austria), and numbered, opaque and sealed envelopes.

Patients were ventilated with the Servoi Ventilator (Maquet Critical Care, Solna, Sweden), and ventilator settings before initiation of the protocol were adjusted by ICU team (Baseline). PEEP (positive end-expiratory pressure) and FIO_2_ (fraction of inspired oxygen) were kept constant during the study. We registered baseline ventilatory parameters and demographic data. Sedation was adjusted by the ICU team according to the ICU sedation protocol, targeting a RASS of − 5 for patients receiving neuromuscular blocker and RASS − 2 to 0 for patients transitioning to assisted ventilation.

**Fig. 1:**
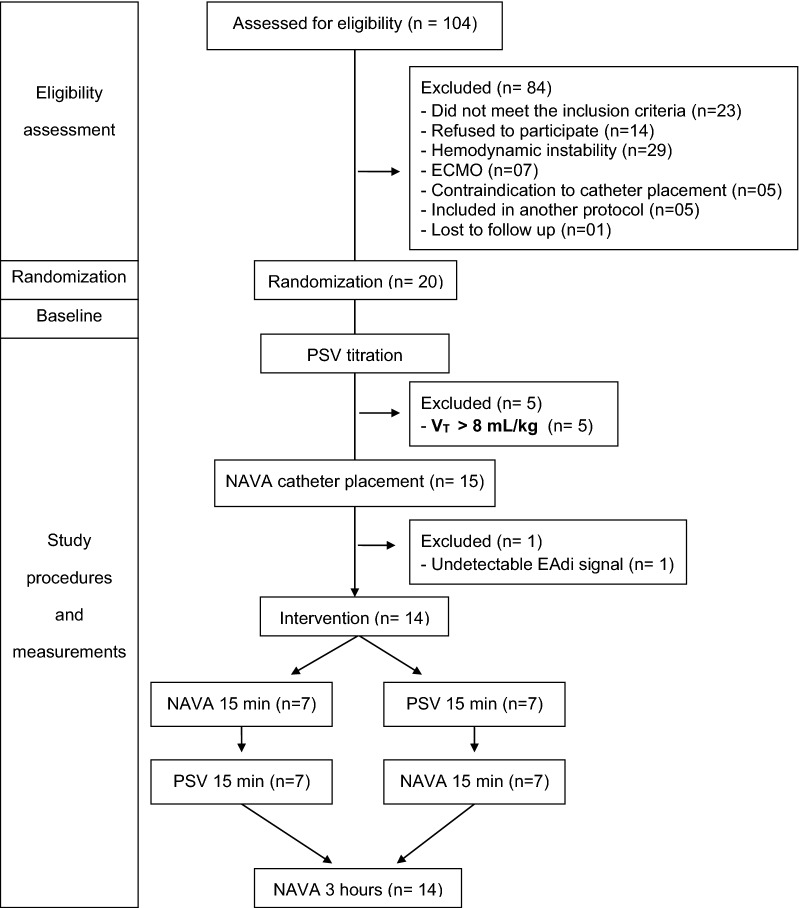
Flow diagram of study patients and procedures. *NAVA* neurally adjusted ventilatory assist, *PSV* pressure support ventilation, *EAdi* electrical activity of the diaphragm

Patients were switched from Baseline to PSV and we titrated PSV to generate a *V*_T_ ≤ 6 mL/kg PBW. We started with a PS level of 10 cmH_2_O and increased or decreased it to reach the target *V*_T_. If *V*_T_ was ≥ 6.5 mL/kg despite using PS ≤ 3 cmH_2_O, the protocol was interrupted, and Baseline was resumed (Fig. 1). If we were able to provide protective MV with PSV, we placed the NAVA catheter and titrated NAVA. Triggering sensitivity and cycling criteria in PSV were adjusted by the ICU team. The ICU team was instructed to use flow triggering adjusted to be as sensitive as possible without generating auto-triggering.

As previously described [30], we measured EAdi using a dedicated NAVA catheter (Maquet, Sweden) and titrated NAVA level to generate the same peak pressure generated with PSV. Triggering sensitivity in NAVA was fixed at 0.5 µV, and cycling criteria was fixed at 70% of peak EAdi. Pneumatic triggering sensitivity was adjusted by the ICU team and kept constant.

Then, patients were ventilated in NAVA and PSV for 15 min each, in the order determined by randomization, followed by ventilation with NAVA for 3 h (NAVA3h).

We collected hemodynamic and respiratory parameters at the end of NAVA and PSV periods and every 15 min in the NAVA3h and obtained arterial blood gases at the end of NAVA and PSV periods and NAVA3h.

We recorded peak airway pressure (Paw), flow, and EAdi continuously from the ventilator at a sampling rate of 100 Hz, using dedicated software (ServoTracker V.4.2; Maquet, Solna, Sweden). We analyzed 3-min recordings of the end of the period of Baseline, NAVA and PSV ventilation. For analysis of NAVA3h, data from several consecutive recordings were processed in order to analyze at least 10 min of recording for every 30 min.

We processed and analyzed the data using a custom computer routine (Matlab, Mathworks, MA, USA), which automatically detected the moment of initiation and termination of inspiratory efforts and ventilator cycles, and calculated *V*_T_, Paw, respiratory rate (RR), minute ventilation, mean airway pressure (MAP), EAdi peak, and neural inspiratory time (TIn): as the difference of time (in seconds) between the initiation of an inspiratory effort and EAdi Peak. We averaged all cycles in each situation to generate a mean value for the above variables. We calculated EAdi/TIn for each respiratory cycle to have an index of respiratory drive.

Asynchrony event detection was based on a custom computer routine (Matlab, Mathworks, MA, USA) which detected the beginning and termination of each ventilator cycle, the beginning and termination of each respiratory effort and calculated cycle times and mean inspiratory times. This analysis was followed by visual inspection of Paw, flow and EAdi waveforms to confirm the presence of asynchrony. We defined the types of asynchrony in accordance with previous publications [31–34] as: auto-triggering, a ventilator cycle not preceded by an inspiratory effort; triggering delay, delay between start of patient effort and triggering > 25% mean inspiratory time; ineffective effort, an inspiratory effort not accompanied by a ventilator cycle; double triggering, two cycles separated by an expiratory time less than half of mean inspiratory time; prolonged cycle, inspiratory time greater than twice the mean inspiratory time; short cycle, inspiratory time less than half the mean inspiratory time.

The asynchrony index (AI) was calculated as the number of cycles with major asynchronies (auto-triggering, ineffective efforts, double triggering and short cycle) divided by the number of monitored neural cycles, expressed as a percentage.

The primary endpoint was *V*_T_ in mL/kg of predictive body weight, and secondary endpoints were Paw, EAdi, RR and asynchrony index.

### Statistical analysis

Continuous variables are expressed by mean and standard deviation or median and 25–75% interquartile range. We used paired t tests to compare continuous variables. For non-normally distributed variables, we used paired Wilcoxon signed-rank tests. Statistical analysis was done with R (http://www.R-project.org/, Vienna, Austria). A *p* value less than 0.05 was considered significant.

## Results

We assessed 104 patients with *P*/*F* ratio less than 300, and excluded 84, mainly because they did not meet the inclusion criteria, refused to participate or had severe hemodynamic instability (Fig. 1). Twenty patients were randomized and included in the trial, and 14 completed the protocol. The protocol was interrupted for five patients (25%) for whom we were unable to maintain *V*_T_ below 6.5 mL/kg PBW in PSV due to strong inspiratory efforts and for one patient for whom we could not detect the EAdi signal.

Patient’s demographic characteristics are shown in Table 1.

**Table 1:** Patient demographics at admission and at diagnosis of ARDS

ID	Age	Gender	SAPS 3	Cause of ARDS	Charlson	*P*/*F*
1	51	M	43	Aspiration	3	102
2	54	F	67	Aspiration	2	136
3	79	M	71	Pneumonia	5	114
4	56	M	27	Pneumonia	2	90
5	63	M	74	Pneumonia	5	120
6	42	M	75	Pneumonia	2	160
7	73	F	52	Pneumonia	4	192
8	60	F	58	Pneumonia	3	162
9	41	M	39	Pneumonia	1	70
10	48	M	46	Pneumonia	1	127
11	79	M	63	Pneumonia	5	175
12	45	F	52	Pneumonia	1	190
13	61	F	96	Anaphylactic shock	4	58
14	40	F	31	Pneumonia	2	139
15	33	F	38	Pneumonia	2	289
16	51	F	73	Pneumonia	1	96
17	46	M	61	Pneumonia	1	194
18	56	M	51	Pneumonia	3	140
19	37	M	70	Pneumonia	6	106
20	46	F	67	Pneumonia	1	140

*ID* patient identification, *Age* age in years, *SAPS 3* Simplified Acute Physiology Score 3 calculated at admission, *ARDS* acute respiratory distress syndrome, *Charlson* Charlson comorbidity index at admission, *P/F* ratio of arterial oxygen pressure divided by the fraction of inspired oxygen on the day of the diagnosis of ARDS

The baseline mode was volume-controlled for six patients and pressure-controlled for 14 patients. Ventilatory settings during the study protocol are shown in Table 2. Most patients received neuromuscular blockage on the first 48 h of the diagnosis, but none were still receiving it at the time of the study. Sedation was used for most patients at ICU team discretion, targeting a RASS of − 2 to 0, but since we studied patients a few hours after NMB interruption, some patients were still deeply sedated. The most commonly sedatives used were fentanyl, propofol and midazolam (Table 2).

**Table 2:** Ventilator parameters and patient characteristics during the study protocol

ID	MV_days_	Mode	PS	Cycl_off_	NAVA_L_	PEEP	FIO_2_	*P*/*F*	Sedation	RASS
1	6	PCV	13	30%	2.4	11	0.45	151	F	− 5
2	7	PCV	NA	NA	NA	16	0.35	183	None	− 2
3	3	PCV	NA	NA	NA	14	0.60	129	F, P	− 4
4	3	PCV	5	30%	0.2	15	0.30	376	F, P	− 4
5	1	PCV	8	25%	1.2	10	0.50	296	None	0
6	3	PCV	10	30%	0.6	10	0.45	136	F	− 4
7	4	PCV	6	40%	1.4	8	0.40	265	F, P	− 4
8	3	VCV	6	35%	0.4	8	0.30	281	F, M	− 5
9	3	PCV	NA	NA	NA	8	0.50	114	F, M, D	+ 1
10	2	PCV	5	30%	0.8	15	0.50	187	F, P	− 4
11	3	PCV	NA	NA	NA	8	0.30	213	P	− 3
12	3	VCV	5	30%	0.6	10	0.30	240	F	− 2
13	3	PCV	7	30%	0.3	10	0.35	193	F, M	− 4
14	3	PCV	6	30%	0.5	10	0.30	280	D	− 1
15	2	VCV	8	30%	2.0	10	0.50	137	F, P, M	− 3
16	4	PCV	12	15%	2.2	10	0.45	170	None	− 3
17	2	VCV	7	30%	0.5	8	0.40	215	F, M	− 4
18	4	VCV	10	30%	0.8	12	1.00	133	F	0
19	5	PCV	NA	NA	NA	13	1.00	314	F, P	− 4
20	4	VCV	NA	NA	NA	10	0.45	149	F, M	− 3

*ID* patient identification, *MV*_*days*_ days of mechanical ventilation before inclusion on the study, *PS* pressure support, *Cycl*_*off*_ cycling off in pressure support mode, *NAVA*_*L*_ level of neurally adjusted ventilatory assist (NAVA) during ventilation with NAVA mode, *PEEP* positive pressure at the end of expiration, *FIO*_*2*_ fraction of inspired oxygen, *P/F* ratio of arterial oxygen pressure divided by the fraction of inspired oxygen on the day of the study, *F* continuous fentanyl, *P* continuous propofol, *M* continuous midazolam, *D* continuous dexmedetomidine, *RASS* Richmond Agitation–Sedation Scale at the day of study, *NA* not applicable

The *V*_T_ stayed within protective levels for the 14 patients who completed the protocol, and there was no difference between NAVA and PSV (Table 3). There was also no statistical difference in RR, minute ventilation, MAP, EAdi and EAdi/TIn comparing the two modes. Paw was greater in NAVA than in PSV, but it remained at protective levels. Median PaO_2_ and median *P*/*F* were greater in NAVA than in PSV. There were no differences for other blood gas variables between NAVA and PSV (Table 4) or in the blood gases comparing baseline and NAVA3h (Additional file 1: Table S1).

**Table 3:** Respiratory variables during the study protocol

Variable	NAVA	PSV	*p* value
RR (rpm)	24 ± 7	23 ± 7	0.661
*V*_T_/kg (mL/kg)	5.8 ± 1.1	5.6 ± 1.0	0.455
VE (L/min)	8.2 ± 2.5	8.0 ± 2.5	0.431
MAP (cmH_2_O)	12.7 ± 2.4	12.5 ± 2.3	0.364
Paw (cmH_2_O)	21 ± 3	19 ± 3	0.001
EAdi (µV)	12.9 ± 6.8	11.9 ± 6.9	0.285
EAdi/TIn (µV/s)	19.3 ± 9.4	17.3 ± 8.8	0.156

*RR* respiratory rate, *V*_*T*_*/kg* tidal volume for predicted body weight, *VE* minute ventilation, *MAP* mean airway pressure, *Paw* peak airway pressure, *EAdi* peak of electrical activity of the diaphragm, *EAdi/TIn* peak of electrical activity of the diaphragm divided for neural inspiratory time − index of respiratory drive

Data presented as mean ± standard deviation, *p* value obtained by paired *t* test

**Table 4:** Blood gases during the study protocol

Variable	NAVA	PSV	*p* value
pH	7.37 (7.36–7.41)	7.37 (7.36–7.40)	0.481
PaO_2_	88 (69–96)	80 (66–96)	0.045
*P*/*F*	241 (203–265)	236 (144–260)	0.050
PaCO_2_	39 (36–44)	40 (37–45)	0.290
HCO_3_	23 (21–24)	23 (21–25)	0.575
BE	− 1.1 (− 2.7 to 0.1)	− 0.1 (− 3.7 to 0.4)	0.906
SaO_2_	95 (92–97)	96 (92–96)	0.861

*pH* hydrogen potential, *PaO*_*2*_ arterial oxygen pressure, *P/F* ratio of arterial oxygen pressure divided by the fraction of inspired oxygen, *PaCO*_*2*_ arterial pressure of carbon dioxide, *HCO*_*3*_ bicarbonate, *BE* base excess, *SaO*_*2*_ oxygen saturation

Data presented as median and 25–75% interquartile range. *p* value obtained by Wilcoxon signed-rank test

**Fig. 2:**
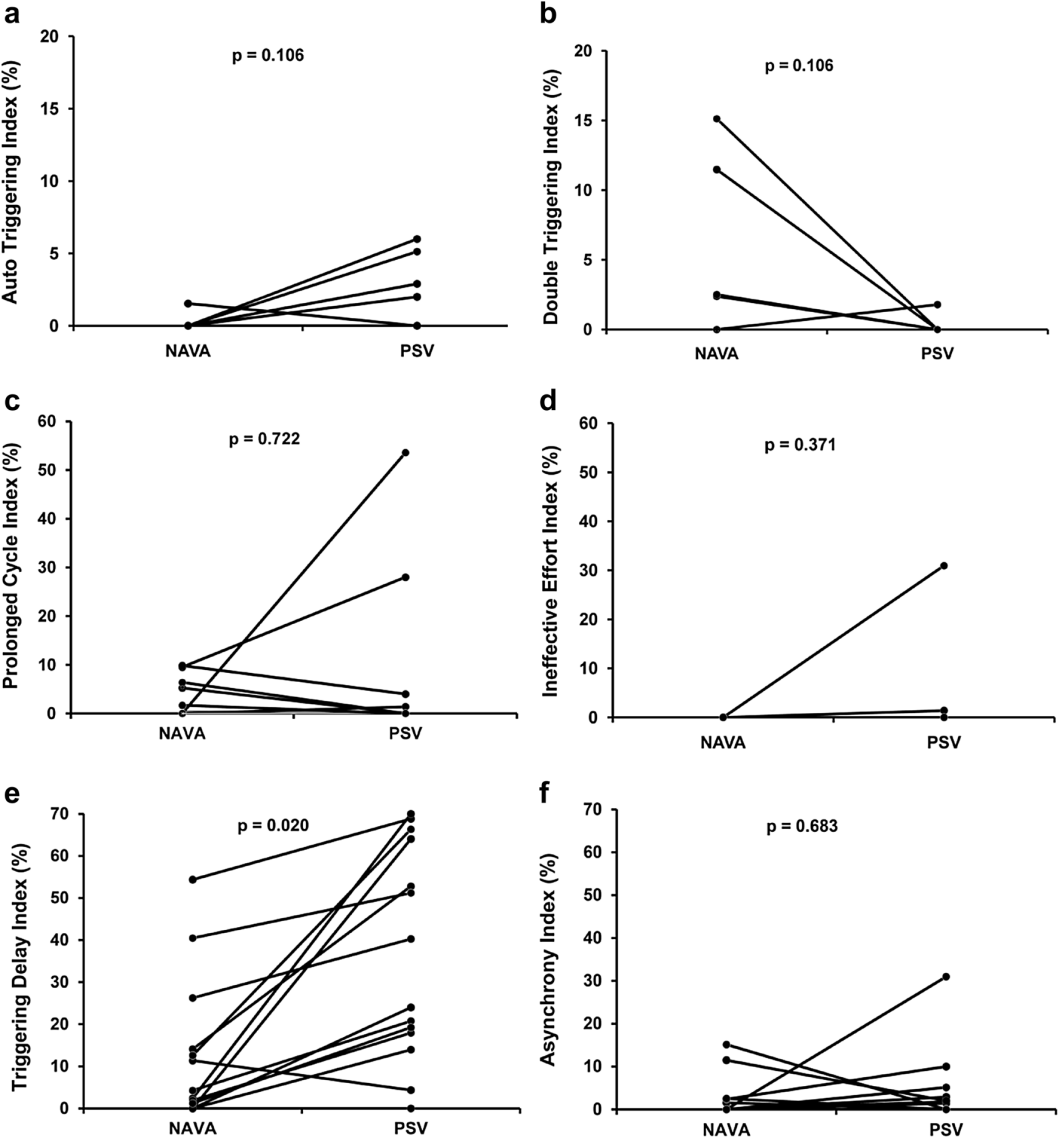
Asynchrony analysis in NAVA and PSV. Lines represent each patient asynchrony index for each type of asynchrony. *NAVA* neurally adjusted ventilatory assist, *PSV* pressure support ventilation. *p* values obtained with the Wilcoxon signed-rank test. The asynchrony index (AI) includes the following major asynchrony types: auto-triggering, ineffective efforts, double triggering and short cycle

**Fig. 3:**
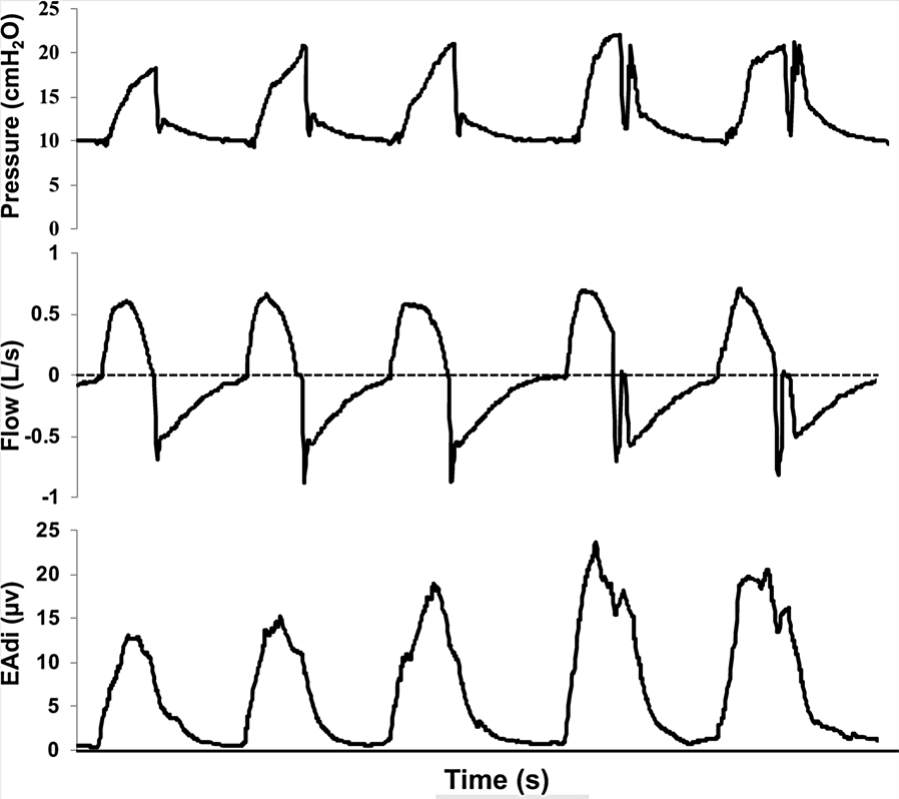
Double triggering in NAVA. Pressure, flow and EAdi vs. time in a representative recording of a patient exhibiting double triggering in NAVA (visible in the last two respiratory cycles). The double triggering was related to EAdi signal showing a biphasic curve. Note that second cycle resulted in an increase in airway pressure, but had zero flow and did not result in breath stacking

Comparisons of the incidence of asynchronies between NAVA and PSV for each patient are shown in Fig. 2. Auto-triggering (Fig. 2a) was similar for both modes; Double triggering (Fig. 2b) was observed in four patients during NAVA and in one patient during PSV, but the difference was not significant. In NAVA, the second cycle in a double triggering event had very low or zero flow (Fig. 3). Prolonged cycle (Fig. 2c) was similar in NAVA and PSV; ineffective effort was observed in two patients during PSV, and absent during NAVA (Fig. 2d). Triggering delay (Fig. 2e) was observed in 13 patients during PSV and in 10 patients during NAVA. Short cycle was observed in only one patient in PSV. AI was greater than 10% in two patients in both PSV and NAVA and was not significantly different between the two modes of ventilation (Fig. 2f), with a median of 0.7% (0–2.7) in PSV and 0% (0–2.2) in NAVA (*p* = 0.6835).

**Fig. 4:**
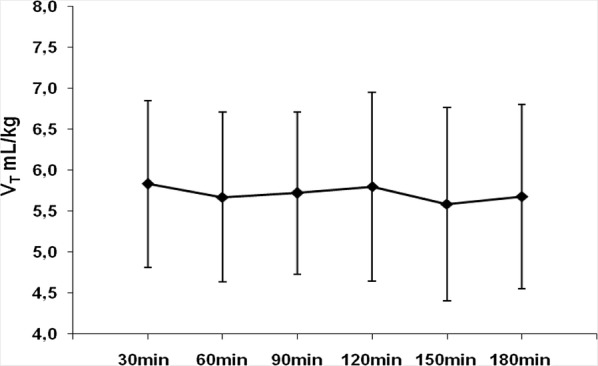
Tidal volume over the 3 h of ventilation with NAVA. Filled diamonds represent the mean tidal volume for predicted body weight of 13 patients who completed the 3 h of NAVA and bars represent standard deviation. *V*_T_ mL/kg: tidal volume for predicted body weight

To evaluate if protective MV could be sustained with NAVA, we compared the *V*_T_, RR and Paw for the 13 patients who completed the period of NAVA3h with controlled ventilation at Baseline. There was no statistical difference in *V*_T_ (5.8 ± 1.2 mL/kg in NAVA and 5.5±0.8 mL/kg in baseline; *p* = 0.364), RR (24 ± 7 in NAVA and 25 ± 7 in baseline; *p* = 0.946), or Paw (20 ± 4 cmH_2_O in NAVA and 23 ± 4 cmH_2_O in baseline; *p* = 0.051). The median *P*/*F* ratio was similar for both modes, 216 (172–281) vs. 217 (167–266), *p* = 0.765. One patient who presented apneas on the first hour of NAVA3h was excluded from this assessment. Mean *V*_T_ was stable and remained at protective levels over NAVA3h (Fig. 4).

## Discussion

In this study, we compared *V*_T_, RR, Paw, gas exchange and patient–ventilator asynchronies in NAVA and PSV in patients with ARDS on the first day after neuromuscular blockage were interrupted. The main findings were: (1) most patients could be ventilated in PSV and NAVA within protective levels, but one-quarter had strong respiratory efforts that resulted in high tidal volumes that prevented the application of protective ventilation with partial ventilatory support. (2) NAVA and PSV resulted in similar *V*_T_ and RR; NAVA resulted in greater Paw than PSV, but Paw in both modes remained within protective levels. (3) There was no difference in the asynchrony index between NAVA and PSV. (4) NAVA was well tolerated during a 3-h period and had similar *V*_T_, Paw and RR compared to controlled ventilation.

To our knowledge, this is the first study using NAVA while providing protective ventilation in ARDS patients. Our protocol began a few hours after the deep sedation and/or neuromuscular blockage was discontinued and patients had respiratory drive. Other studies using NAVA in patients with ARDS were performed in the weaning phase [25, 34], in patients on ECMO [33] or did not specify timing [24]. The largest trial using NAVA randomized 128 patients to NAVA or PSV in the beginning of the weaning of MV, but the study population was a mix of several causes of respiratory failure [35]. Our intention was to apply partial ventilatory support—NAVA and PSV—while providing protective MV with a potential to reduce the need for sedation and the occurrence of asynchrony, as an improved strategy to avoid ventilator-induced lung injury (VILI) [36].

We found that this approach was feasible for most ARDS patients under continuous sedation, and a short trial on PSV was used to detect patients with strong inspiratory efforts who could not be kept on protective ventilation in partial ventilatory support. Patients who could be ventilated with approximately 6 mL/kg in PSV could also be safely ventilated in NAVA. We included patients on the three categories of ARDS, with a median of 3 days of MV. On the day of the study, the median *P*/*F* was still low, 190, and the two patients with *P*/*F* above 300 had received recruitment maneuvers and were on high PEEP (13 and 15 cmH_2_O). Tidal volume was similar in PSV and NAVA and within protective values for the 14 patients who completed the protocol. However, we had to interrupt the protocol and resume deep sedation for five patients (25%) who had strong inspiratory efforts resulting in *V*_T_ ≥ 8 mL/kg, even with low levels of PS. Our concern was that strong inspiratory efforts and high tidal volumes could contribute to VILI [37]. In such cases, patients may benefit from the use of controlled ventilation and sedation to avoid further injury caused by MV [6].

The breathing pattern was similar in NAVA and PSV, there were no differences in RR, in accordance with previous studies [24, 33]. The respiratory drive was similar in both modes for the 14 patients enrolled, as indicated by the mean values of EAdi/TIn and EAdi. Paw was greater in NAVA than in PSV, despite titration of NAVA level to generate the same Paw in PSV. That happened because Paw in NAVA varies with EAdi, in contrast to PSV, where it is set. However, Paw remained within protective levels in both modes and this difference did not translate into more assistance in NAVA compared to PSV, since RR, *V*_T_ and EAdi were similar in both modes [21, 38, 39]. The *P*/*F* ratio was higher in NAVA when compared with PSV, but the difference was not clinically relevant.

The incidence of the asynchrony was relatively low in our study. AI was greater than 10% in only two patients in both PSV and NAVA, which is considered clinically important and associated with prolonged mechanical ventilation [31, 32, 40]. This finding may be related to the fact that most patients were still sedated during the study and that they were ventilated with low tidal volumes, which may have prevented the occurrence of ineffective triggering [41] the most common type of asynchrony [40, 42].

Triggering delay was the most common type of asynchrony in our study, and it was significantly reduced in NAVA compared to PSV. Since triggering delay greater than 0.15 s may cause considerable discomfort [26, 43] and is easily measured with the NAVA catheter [44–46], we report its value but do not include it in the computation of AI to allow comparison of our results with other studies.

The occurrence of ineffective effort was rare and observed in only two patients in PSV. This is a contrast with previous studies comparing NAVA and PSV [22, 23, 25, 26, 45]. One possible explanation for this difference is that the most important risk factor for ineffective effort is over assistance and air trapping. Since ARDS patients have low respiratory system compliance and we titrated NAVA and PSV to deliver low *V*_T_, risk factors for air trapping were reduced.

Double triggering was more common in NAVA than in PSV. This result is consistent with results obtained in other studies [26, 47]. The presence of double triggering in NAVA is related to EAdi signal showing a biphasic curve. In our study, the second cycle in NAVA usually resulted in zero flow and did not result in breath stacking and high *V*_T_, which is a concern related to double triggering in assisted-controlled modes [24, 48–50]. Auto-triggering was observed in four patients in PSV and in only one patient during NAVA.

The low incidence of prolonged cycle compared to previous studies [22, 23, 25, 26] is related to patients’ mechanics and influenced by the cycling criterion used. In NAVA, the cycling criterion is fixed at 70% of peak EAdi, and in PSV it was adjusted by the ICU team.

NAVA marginally improved PaO_2_ and *P*/*F* compared with PSV, but the difference was not clinically relevant. Previous studies showed that spontaneous breathing efforts are associated with improved gas exchange compared with controlled ventilation [24, 51, 52], but we did not observe differences in gas exchange comparing NAVA3h and controlled ventilation. We found that NAVA was well tolerated for 3 h, and had similar RR, *V*_T_, Paw and hemodynamics compared to controlled ventilation.

### Limitations

Our study has several limitations: first, it was a pilot, single-center study, we recruited a small sample size and patients included in the study showed a great variability regarding the number of days of MV, PEEP levels and *P*/*F* ratio. In addition, each ventilatory mode was studied for a short period. Therefore, more studies are necessary to test the feasibility and safety of using NAVA continuously in patients with ARDS to deliver protective MV. Second, we opted to interrupt the protocol for 25% of patients due to strong inspiratory efforts that prevented us from maintaining low *V*_T_ in PSV. This choice was made to address a safety issue, since there is uncertainty about deleterious effects of strong inspiratory efforts at the early phase of ARDS. Since the patients had already been randomized, our power to detect differences between NAVA and PSV was reduced. Therefore, our conclusions about the safety of NAVA and PSV to deliver protective MV cannot be extrapolated to all patients with ARDS. And third, we used the EAdi to identify patients’ inspiratory efforts and asynchrony events; therefore, inspiratory efforts initiated by accessory muscles were not detected, which might impact asynchrony detection. In addition, EAdi is a processed signal, which may have impacted precision.

## Conclusions

In this pilot study, we found that it was feasible to keep tidal volume within protective levels with NAVA and PSV for 75% of the patients with ARDS under continuous sedation. Tidal volume was similar in PSV and NAVA and remained within protective levels for 3 h with NAVA. These findings suggest that using partial ventilatory assistance in ARDS can be used as part of a protective ventilation strategy, with potential benefits of less sedation and less muscle paralysis.

## Supplementary information

**Additional file 1: Table S1.** Comparison of blood gases between controlled ventilation and NAVA at the end of 3 h.

## Data Availability

All data are available in the manuscript.

## Abbreviations

AI: Asynchrony index
ARDS: Acute respiratory distress syndrome
EAdi: Electrical activity of the diaphragm
EAdi peak: Peak of electrical activity of the diaphragm
ECMO: Extracorporeal membrane oxygenation
FIO_2_: Fraction of inspired oxygen
ICU: Intensive care unit
MAP: Mean airway pressure
MV: Mechanical ventilation
NAVA: Neurally adjusted ventilatory assist
NAVA3H: Neurally adjusted ventilatory assist delivered for three hours
Paw: Peak airway pressure
PBW: Predicted body weight
PEEP: Positive end-expiratory pressure
*P*/*F*: Ratio of arterial oxygen pressure divided by the fraction of inspired oxygen
Pplat: Plateau pressure
PSV: Pressure support ventilation
RASS: Richmond Agitation–Sedation Scale
RR: Respiratory rate
SAPS 3: Simplified acute physiology score
TIn: Neural inspiratory time
VE: Minute ventilation
*V*_T_: Tidal volume
