# Supplementary material for: Neurally adjusted ventilatory assist vs. pressure support to deliver protective mechanical ventilation in patients with acute respiratory distress syndrome: a randomized crossover trial
Source: Ann Intensive Care. 2020 Feb 10;10:18. doi: 10.1186/s13613-020-0638-0 (PMC7010869; doi:10.1186/s13613-020-0638-0)
Supplement: Supplementary file 1 — Additional file 1: Table S1. Comparison of blood gases between controlled ventilation and NAVA at the end of 3 h. [file 13613_2020_638_MOESM1_ESM.docx]

Table S1: Comparison of blood gases between controlled ventilation

and NAVA at the end of three hours

| **Variable** | **NAVA 3h** | **CMV** | **p value** |
| --- | --- | --- | --- |
| pH | 7.39 (7.38-7.40) | 7.39 (7.37-7.42) | 0.384 |
| PaO_2_ | 78 (72-83) | 76 (68-85) | 0.894 |
| P/F | 217 (167-266) | 216 (172-281) | 0.765 |
| PaCO_2_ | 39 (36-43) | 37 (36-41) | 0.756 |
| HCO_3_ | 23 (22-24) | 24 (22-24) | 0.248 |
| BE | -1.0 (-2.5-0.2) | -0.16(-2.4-0.5) | 0.320 |
| SaO_2_ | 94 (93-96) | 95 (93-97) | 0.266 |

CMV: controlled mechanical ventilation; pH: Hydrogen potential; PaO_2_: arterial oxygen pressure; P/F: ratio of arterial oxygen pressure divided by the fraction of inspired oxygen; PaCO_2_: arterial pressure of carbon dioxide; HCO_3_: Bicarbonate; BE: base excess; SaO_2_: oxygen saturation. Data presented as median and 25-75% interquartile range. p-value obtained by Wilcoxon Signed-rank Test.
